# Efficacy of acupuncture for functional constipation in elderly: a systematic review and meta-analysis

**DOI:** 10.3389/fmed.2024.1473847

**Published:** 2024-12-04

**Authors:** ShiWei Song, WenFeng Hao, HongFang Fu

**Affiliations:** ^1^Department of Traditional Chinese Medicine, Sichuan Taikang Hospital, Chengdu, China; ^2^Department of Traditional Chinese Medicine, Sichuan Cancer Hospital & Institute, Sichuan Cancer Center, Affiliated Cancer Hospital of University of Electronic Science and Technology of China (UESTC), Chengdu, China

**Keywords:** acupuncture, functional constipation, elderly people, meta-analysis, systematic review

## Abstract

**Background:**

Numerous clinical studies have shown that patients suffering from functional constipation can benefit by combining medication with acupuncture. There have been no published reviews or meta-analyses regarding the use of acupuncture in treating functional constipation in older adults. Therefore, we carried out a meta-analysis to assess the impact of acupuncture on elderly patients dealing with functional constipation.

**Methods:**

This study retrieved randomized controlled trials (RCTs) on acupuncture therapy for functional constipation in the elderly from several electronic databases, including Embase, PubMed, Cochrane Library, Wanfang Database, Chinese BioMedical Literature Database, and China National Knowledge Infrastructure. In these databases, clinical investigators evaluated the effectiveness of acupuncture as a primary treatment for elderly people with functional constipation. *The Cochrane Handbook for Systematic Reviews of Interventions* was used by researchers to evaluate the quality of the study.

**Results:**

A total of 469 elderly individuals were included in 8 RCTs. The meta-analysis yielded compelling findings: the application of acupuncture has significantly elevated the rates of treatment effectiveness compared to the control group. Compared with the control group, the treatment group exhibited a statistically significant difference in complete spontaneous bowel movements after treatment. The two groups showed no significant difference in spontaneous bowel movements. However, there was a significant difference in the Bristol Stool Scale scores. The Defecation Difficulty Score and Patient Assessment of Constipation Quality of Life (PAC-QOL) showed *p*-values that indicated no significant effect. However, acupuncture improved bowel movements, demonstrating a significant difference in the Clinic Constipation Score (CCS) when comparing the two groups. The Nitric Oxide Synthase (NOS) and 5-Hydroxytryptamine (5-HT) contents changed significantly after intervention in both groups. An article reported that the improvement of traditional Chinese medicine (TCM) symptom scores was better in the treatment group than in the control group.

**Conclusion:**

The analysis results indicated that acupuncture can be beneficial for elderly people with functional constipation; however, strong and comprehensive data are not yet obtainable. Given that our study is based on evidence that is of a low-to-moderate quality, further high-quality research is necessary to enhance the feasibility and practicability of this treatment.

**Systematic review registration:**

https://www.crd.york.ac.uk/PROSPERO/, identifier CRD42024533215.

## 1 Introduction

Functional constipation represents a prevalent clinical condition (1), often manifesting not only as a standalone ailment but also as a symptomatic expression of other underlying health disorders (2, 3). Globally, the incidence of constipation among adults varies widely, with studies indicating a range from 2.5 to 79.0%. In developed countries such as the United States, the United Kingdom, and Canada, a significant proportion of the healthy population estimated at 10–15% is affected by functional constipation. In China, the prevalence of constipation among adults is relatively lower, with an estimated range of 4–6%. However, this figure escalates with advancing age, highlighting a demographic trend that is particularly concerning (4). Among those in the senior population, aged 60 and above, the prevalence of constipation soars to a staggering 22% (5). Moreover, this percentage is on an upward trajectory, reflecting an emerging public health challenge that requires increased attention and proactive measures to address the needs of this vulnerable age group. The primary symptoms are defecation less than three times per week, hard dry stool, or difficulty in defecating (6, 7). Most patients have recurrent symptoms and are plagued by disease for a long time, which will affect patients’ normal lives, work, and studies (8). Long-term treatment for chronic conditions can impose substantial economic and psychological strains on patients and their families (9). In severe cases, it may induce cardiovascular and cerebrovascular diseases as well as psychological disorders (10). The common therapeutic strategies for managing functional constipation encompass a spectrum of interventions, ranging from lifestyle and dietary adjustments to the use of bulking agents, stool softeners, osmotic and stimulant laxatives, as well as prokinetic agents (11, 12). While these conventional treatments can provide temporary symptomatic relief, it is important to note that they are accompanied by certain side effects (13). Consequently, there has been a growing trend toward the adoption of complementary and alternative therapies, with acupuncture being a notable example (14, 15).

Numerous clinical studies in recent years have shown that the use of acupuncture has unique advantages in the treatment of this disease (14). Acupuncture is used not only for functional constipation but also for drug-related constipation and oncological constipation, with documented success. Both conventional manual acupuncture and electroacupuncture can significantly improve the symptoms of patients, reduce inflammation, and improve the quality of life of patients (16, 17).

However, to date, no systematic reviews have specifically addressed the application of acupuncture for treating functional constipation in the elderly population. Meanwhile, previous studies, due to insufficient blinding methods, small sample sizes, and lengthy periods of research data, have mainly focused on constipation in adults, neglecting the related research on functional constipation in the elderly. At the same time, the treatment methods in previous studies were not comprehensive enough, often combining other methods to treat functional constipation, which does not fully reflect the research achievements of recent years. Recognizing this gap in the literature, we undertook this meta-analysis to critically evaluate the efficacy of acupuncture in alleviating functional constipation among the elderly.

We aim to contribute a solid evidence-based framework that can inform clinical practice and guide healthcare decisions. At the same time, based on the existing scientific literature and theoretical foundations, we will propose that acupuncture may work by stimulating intestinal motility or regulating gastrointestinal neurotransmitters. Through our research, we hope to provide more ideas and directions for the mechanism study of acupuncture in treating functional constipation in the elderly population.

## 2 Materials and methods

This meta-analysis fully complies with the Preferred Reporting Items for Systematic Reviews and Meta-analysis (PRISMA) guidelines. A detailed protocol for the study was registered at PROSPERO (No. CRD42024533215).

### 2.1 Inclusion criteria for literature

1. The literature type was published RCTs on acupuncture treatment of functional constipation in elderly people.
2. Elderly patients who were diagnosed with functional constipation according to the Rome II/III criteria for chronic functional constipation and severe chronic constipation, regardless of gender, race, color of skin, and nationality.
3. In the literature, the experimental group received acupuncture treatments, while the control group received routine treatments for functional constipation.
4. The language of publication was limited to English and Chinese.

### 2.2 Literature exclusion criteria

1. Studies without diagnostic criteria for functional constipation.
2. Studies of manual acupuncture (MA) or electroacupuncture (EA) combined with other therapies.
3. Repeatedly published literature.
4. Research on animal experiments, case reports, letters, reviews, and experience summaries of doctors.
5. Defective literature data.
6. Impractical full-text and outcomes.
7. Nonclinical randomized controlled trials.

### 2.3 Search strategy

The following databases were searched from the inception of the databases to July 23, 2024: PubMed, Cochrane Library, Embase, China National Knowledge Infrastructure, China Biomedical Literature Database, and Wanfang Database. In addition, gray literature was searched manually. The search keywords included “constipation,” “functional constipation,” “pharmacopuncture,” acupuncture,” “acupuncture treatment,” “acupuncture therapy,” “needling therapy,” “randomized controlled trial,” and “clinical trials.” The study selection process of PubMed is taken as an example, which is shown in Table 1.

**Table 1:** PubMed: session results.

Number	Query	Search details	Results
#8	#3 AND #6	((“Constipation”[MeSH Terms] OR “Constipation”[Title/Abstract]) AND (“Acupuncture”[MeSH Terms] OR (“Pharmacopuncture”[Title/Abstract] OR “acupuncture treatment”[Title/Abstract] OR “needling”[Title/Abstract] OR (“acupunture”[All Fields] AND “therapy”[Title/Abstract]) OR “needling therapy”[Title/Abstract]))) AND (clinicaltrial[Filter] OR randomizedcontrolledtrial[Filter])	17
#7	#3 AND #6	(“Constipation”[MeSH Terms] OR “Constipation”[Title/Abstract]) AND (“Acupuncture”[MeSH Terms] OR (“Pharmacopuncture”[Title/Abstract] OR “acupuncture treatment”[Title/Abstract] OR “needling”[Title/Abstract] OR (“acupunture”[All Fields] AND “therapy”[Title/Abstract]) OR “needling therapy”[Title/Abstract]))	67
#6	#4 OR #5	“Acupuncture”[MeSH Terms] OR (“Pharmacopuncture”[Title/Abstract] OR “acupuncture treatment”[Title/Abstract] OR “needling”[Title/Abstract] OR (“acupunture”[All Fields] AND “therapy”[Title/Abstract]) OR “needling therapy”[Title/Abstract])	9,594
#5	((((Pharmacopuncture[Title/Abstract]) OR (Acupuncture treatment[Title/Abstract])) OR (needling[Title/Abstract])) OR (acupunture therapy[Title/Abstract])) OR (needling therapy[Title/Abstract])	“Pharmacopuncture”[Title/Abstract] OR “acupuncture treatment”[Title/Abstract] OR “needling”[Title/Abstract] OR (“acupunture”[All Fields] AND “therapy”[Title/Abstract]) OR “needling therapy”[Title/Abstract]	7,886
#4	“Acupuncture”[Mesh]	“Acupuncture”[MeSH Terms]	2,094
#3	#1 OR #2	“Constipation”[MeSH Terms] OR “Constipation”[Title/Abstract]	36,058
#2	Constipation[Title/Abstract]	“constipation”[Title/Abstract]	31,698
#1	“Constipation”[Mesh]	“Constipation”[MeSH Terms]	16,709

### 2.4 Outcome assessment indicators

The main outcome measures were effective rate and complete spontaneous bowel movements (CSBMs) and spontaneous bowel movements (SBMs).

The secondary outcome assessment indicators were (1) traditional Chinese medicine (TCM) symptom scores; (2) Bristol Stool Scale score; (3) Defecation Difficulty Score; (4) Patient Assessment of Constipation Quality of Life (PAC-QOL); (5) Clinic Constipation Score (CCS); (6) plasma NOS; (7) plasma 5-HT; and (8) changes in bowel movements.

### 2.5 Literature screening and data extraction

Two researchers conducted an independent and meticulous extraction of literature data based on the predefined inclusion and exclusion criteria, subsequently cross-verifying their findings to ensure accuracy. In instances of divergence, discrepancies were addressed through collaborative discussion, with the pursuit of a consensus guiding the resolution process. Should the need arise, the expertise of a third party would be sought to facilitate consensus. The following data of the included literature were extracted by Excel: the first author^’^s name; year of publication; the number of cases; sample size; gender; average age; duration of disorder; treatment period; outcome(s); follow-up duration; adverse reaction and each risk of bias assessment element in RCTs.

### 2.6 Risk bias and quality assessment

The quality of the eight literature studies was evaluated by two reviewers independently using the Review Manager 5.4 software risk bias assessment tool provided by the Cochrane Collaboration RCT risk of bias assessment tool. Evaluation indicators include: (l) sequence generation (selection bias); (2) allocation concealment (selection bias); (3) blinding of patients (performance bias); (4) blinding of outcome measurements (detection bias); (5) incomplete outcome data (attrition bias); (6) elective reporting (reporting bias); (7) other bias. Each indicator contains three levels: low risk, unclear, and high risk.

### 2.7 Statistical analysis

The meta-analysis was conducted utilizing the Review Manager 5.4 software. For the analysis of dichotomous variable data, the odds ratio (OR) was selected as the statistical measure, while continuous outcomes were evaluated using the mean difference (MD), accompanied by the calculation of 95% confidence interval (CI) to quantify the precision of the estimates. For the assessment of heterogeneity, Cochran’s *Q*-test was employed, with a *p*-value of ≤0.10 indicating a significant level of heterogeneity. In conjunction, the *I*^2^ statistic was utilized, where an *I*^2^ value exceeding 50% was indicative of the presence of heterogeneity. When significant heterogeneity was detected (*p* < 0.1, *I*^2^ > 50%), a random-effects model was selected to account for variability across studies. Conversely, a fixed-effects model was applied for a more streamlined analysis. The forest plot and funnel plot indicated the result and evaluated the publication bias of the literature. When necessary, we check the robustness of the results by eliminating low-quality trials.

## 3 Results

### 3.1 Literature search results

**Figure 1:**
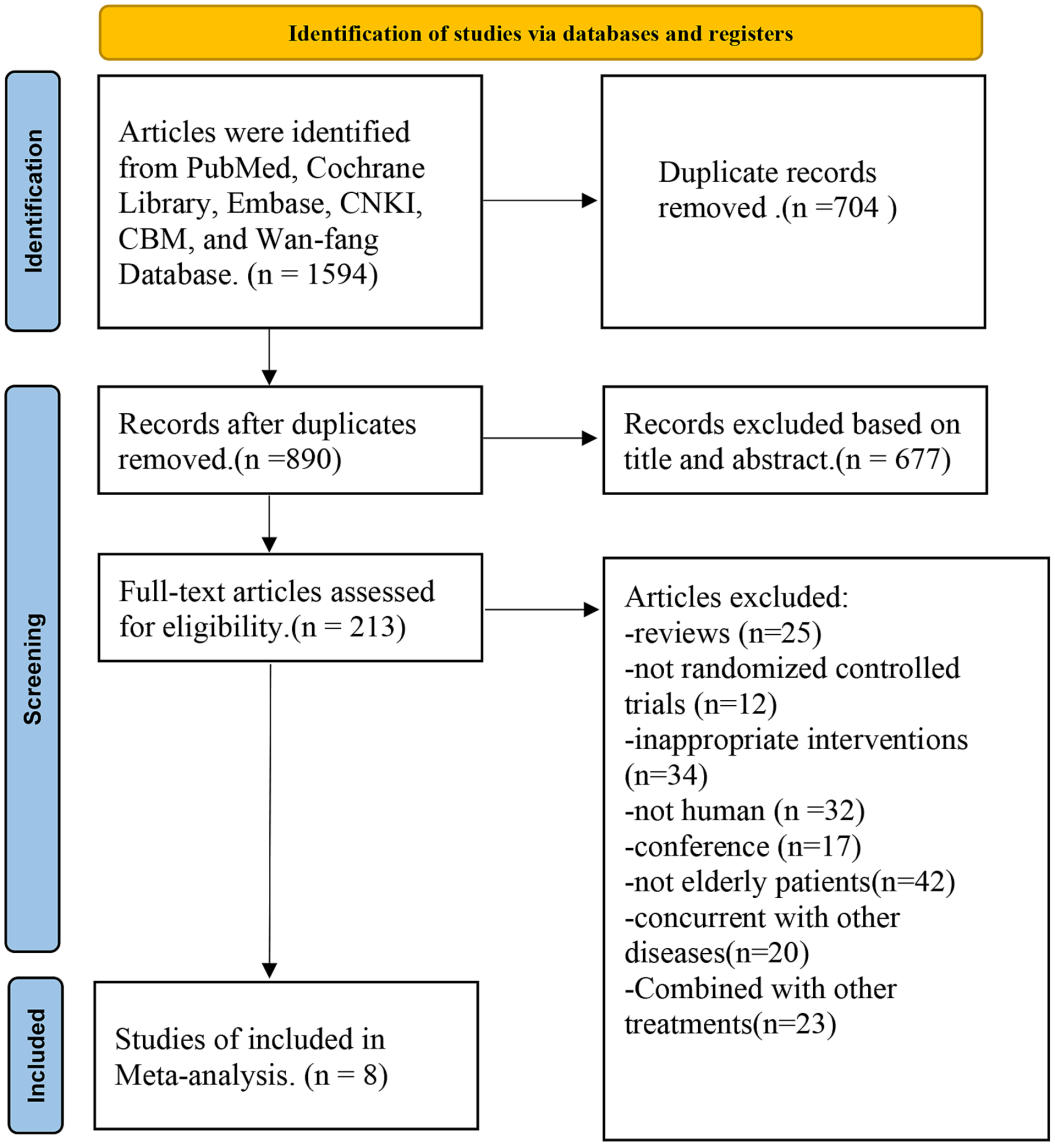
The procedure of literature search and study selection for the research.

According to the search strategy, a total of 1,594 articles were searched, out of which 704 duplicate references were excluded, 677 irrelevant articles were excluded by reading the title and abstract, and 213 articles were retrieved for further evaluation. A total of 205 articles excluded included the following literature: reviews (*n* = 25); not randomized controlled trials (*n* = 12), inappropriate interventions (*n* = 34), not humans (*n* = 32), conferences (*n* = 17), not elderly patients (*n* = 42), and concurrent with other diseases (*n* = 20), combined with other treatments (*n* = 23). Finally, a total of eight studies that met the standards were selected in the meta-analysis (Figure 1).

The general information and characteristics of the eight studies are shown in Table 2 (18–25). A total of eight RCTs were included in this study. The publication period is between 2012 and 2024. A total of 469 patients were enrolled; there were 235 patients in the control group and 234 patients in the treatment group, all of whom were elderly patients with functional constipation. The number of cases ranged from 40 to 80. In the eight studies, the experimental groups selected conventional manual acupuncture or electroacupuncture in the treatment of patients with functional constipation; the control group was treated only with other medications. The control group chose different drugs. The treatment period of eight studies ranged from 8 to 56 days. All studies did not mention the follow-up duration after treatment. Basic information about the literature we selected included: the first author’s name; year of publication; the number of cases; sample size; gender; average age; duration of disorder; treatment period; outcome(s); and follow-up duration.

**Table 2:** Characteristics of eight studies.

Study	Study design	Sample size (T: C)	Age (year), mean ± SD	Disease duration (year), mean ± SD	Sex (M: F)	Intervention	Comparison	Treatment duration (days)	Follow-up (months)	Outcomes
Mao and Zhang (18)	RCT	T: 20 C: 20	T: 62.85 ± 2.71 C: 56.95 ± 9.83	T: 3.69 ± 2.42 C: 3.90 ± 2.75	(T)6: 14 (C)8: 12	Electro-acupuncture	Prucalopride succinate tablets	56	NM	CSBMs, SBMs, Bristol Stool Scale score, Defecation Difficulty Score
Cao (19)	RCT	T: 21 C: 20	T: 51.2 ± 7.23 C: 52.4 ± 7.65	T: 9.83 ± 6.21 C: 10.2 ± 9.7	(T)9: 12 (C)8: 12	Electro-acupuncture	Senna leaf	17	NM	The effective rate
Xu and Zhang (20)	RCT	T: 30 C: 30	T: 53 ± 15 C: 53 ± 12	T: 3.79 ± 3.64 C: 4.03 ± 3.28	(T)12: 18 (C)11: 19	Electro-acupuncture	Sham-electroacupuncture	28	NM	The effective rate, the Clinic Constipation Score, plasma NOS, plasma 5-HT
Hu et al. (21)	RCT	T: 24 C: 25	T: 52.75 ± 18.9 C: 50.96 ± 14.8	T: 12.23 ± 11.98 C: 13.61 ± 14.07	(T)9: 15 (C)6: 19	Electro-acupuncture	Sham-electroacupuncture	28	NM	CSBMs, PAC-QOL
Yan et al. (22)	RCT	T: 29 C: 30	T: 62.79 ± 9.77 C: 59.73 ± 8.49	T: 149.59 ± 109.89 C: 1 32.80 ± 96.05	(T)0: 29 (C)0: 30	Acupuncture	Sham-acupuncture	16	NM	SBMs, Bristol Stool Scale score, Defecation Difficulty Score, PAC-QOL
Li et al. (23)	RCT	T: 40 C: 40	T: 58.06 ± 12.12 C: 57.78 ± 11.54	T: 7.19 ± 5.96 C: 47.7 ± 11.54	(T)18: 22 (C)19: 21	Electro-acupuncture	Senna leaf	40	NM	Changes in bowel movements
Sun et al. (24)	RCT	T: 40 C: 40	T: 60.1 ± 12.4 C: 61.1 ± 13.6	T: 1.8 ± 0.6 C: 1.7 ± 0.8	(T)15: 25 (C)12: 28	Acupuncture	Mosapride citrate dispersible tablets	8	NM	The effective rate, traditional Chinese medicine (TCM) symptom scores
Fu and Yan (25)	RCT	T: 30 C: 30	T: 74.3 ± 2.71 C: 73.5 ± 7.1	T: 6.8 ± 4.2 C: 6.5 ± 0.7	(T)12: 18 (C)13: 17	Acupuncture	Maren Runchang Wan	20	NM	The effective rate

T, intervention group; C, comparison group; M, male; F, female; NM, not mention; RCT, randomized controlled trial; CSBMs, complete spontaneous bowel movements; SBMs, spontaneous bowel movements; PAC-QOL, Patient Assessment of Constipation Quality of Life.

All included studies have comparable baseline characteristics. All included elderly patients with functional constipation who had not received any other treatments before this study.

Whereas four studies assessed the effective rate of treatment, three articles analyzed complete spontaneous bowel movements, two articles measured spontaneous bowel movements, two articles analyzed the Bristol Stool Scale score, two articles analyzed the Defecation Difficulty Score, two articles measured PAC-QOL, only one article analyzed the Clinic Constipation Score, one article analyzed plasma NOS and plasma 5-HT, one article analyzed changes in bowel movements, and one article analyzed the traditional Chinese medicine symptom scores.

### 3.2 Methodological and reporting quality

**Figure 2:**
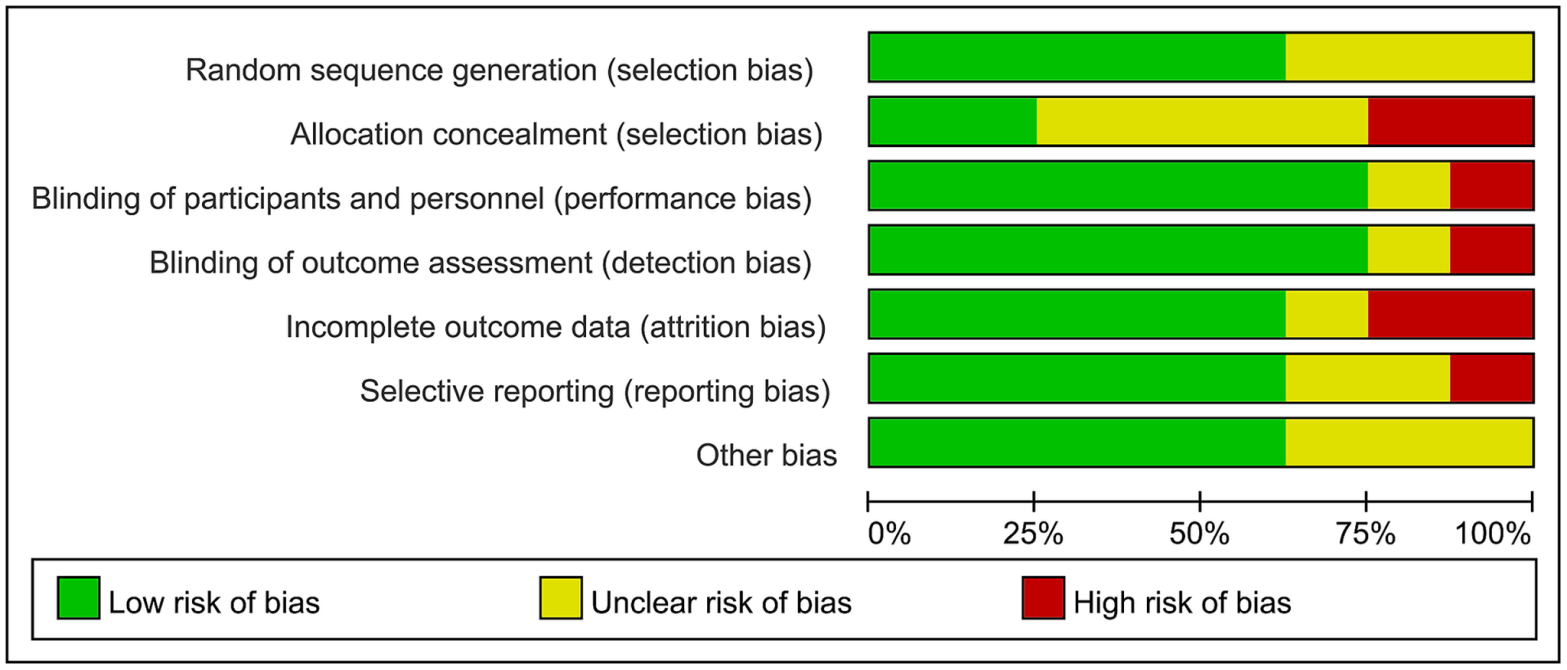
Assessment of the risk of bias of eight articles.

**Figure 3:**
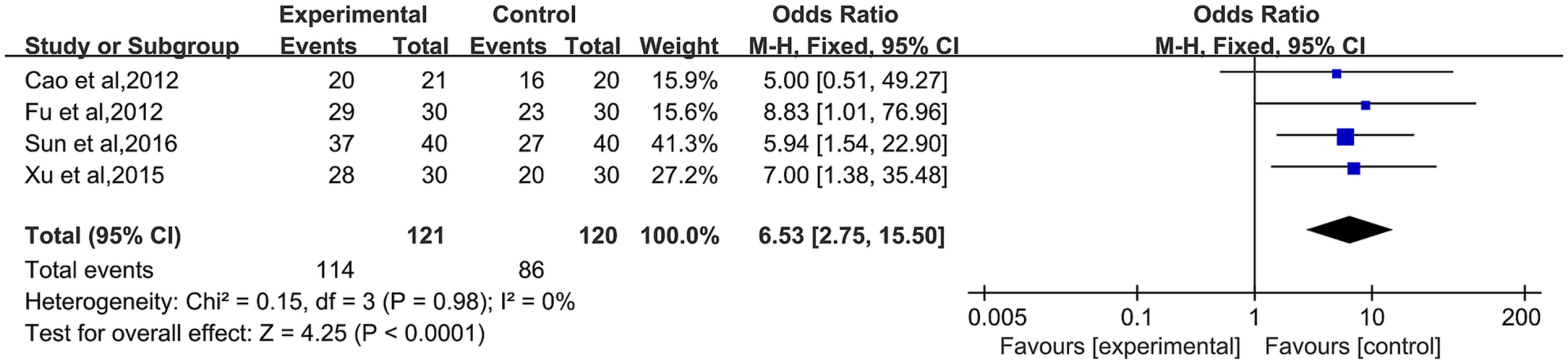
The overall risk bias assessment chart of the eight literature studies.

The included literature was analyzed according to the risk assessment tool. The result showed that the quality of the eight included RCTs was generally“low to moderate.” Five studies were at low risk in terms of random sequence generation. Three studies used random number tables, and the random draw method was used in three studies; only two studies mentioned the randomness. As regards concealment aspects of the allocation schemes, one-fourth of the studies described whether the studies implemented allocation concealment or specific schemes. Blindness: six studies described how the researchers or subjects were blinded. In terms of blind evaluation of outcomes, six articles were at low risk. In terms of the completeness of outcome data, five studies were at low risk. In terms of the risk assessment for reporting bias, only one study was at high risk. In terms of other biases, five articles were at low risk. A summary and graph of the risk of bias assessment are shown in Figures 2, 3.

### 3.3 Effective rate

**Figure 4:**
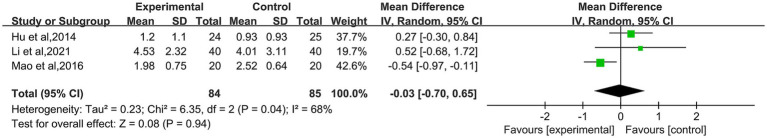
Forest plot of acupuncture efficiency for functional constipation.

About the effective rate of acupuncture in the treatment of functional constipation in old people, four studies (19, 20, 24, 25) were reported. The homogeneity between studies was found to be excellent, so we selected a fixed-effects model (*I*^2^ = 0%; *p* = 0.98). Regarding the effective rate, the experimental group to treat old patients with functional constipation is better than that in the control group (OR: 6.53; 95% CI: 2.75–15.50; *p* < 0.0001, Figure 4).

### 3.4 Complete spontaneous bowel movements

**Figure 5:**
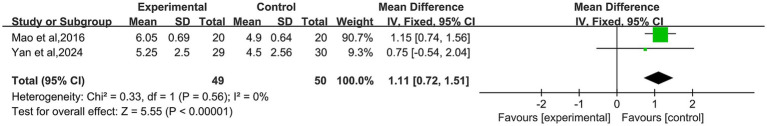
Forest plot of meta-analysis on CSBMs for functional constipation.

In the included studies, a total of three studies (18, 21, 23) recorded CSBMs. A total of 169 old people were treated with moderate heterogeneity (*I*^2^ = 68%; *p* = 0.04). A random-effects model was adopted to analyze the CSBMs (MD: −0.03; 95% CI: −0.70 to −0.65; *p* = 0.94, Figure 5), indicating that acupuncture can better improve the complete spontaneous bowel movements of functional constipation in old people compared with drugs alone.

### 3.5 Spontaneous bowel movements

**Figure 6:**
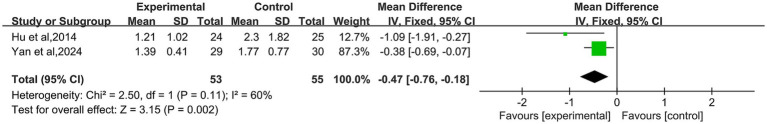
Forest plot of SBMs with acupuncture treatment.

Two types of research with 99 elderly people evaluated spontaneous bowel movements (18, 22). The analysis adopted fixed-effect analysis (MD: 1.11; 95% CI: 0.72–1.51; *p* < 0.00001, Figure 6). No heterogeneity among the studies (*p* = 0.56, *I*^2^ = 0%) were observed. This suggests that acupuncture shows no significant difference compared to the control group in SBMs.

### 3.6 Bristol Stool Scale score

**Figure 7:**
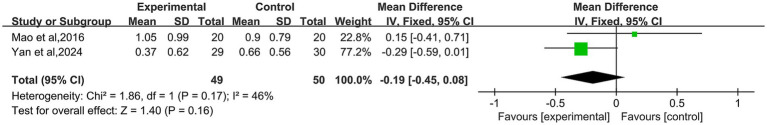
Forest plot of Bristol Stool Scale score of acupuncture for functional constipation in elderly people.

Two studies selected the Bristol Stool Scale score (18, 22). Heterogeneity analysis assessed no significant homogeneity of the studies (*p* < 0.00001, *I*^2^ = 97%). The random-effects analysis was applied to inspect two groups of samples (MD: −0.51; 95% CI: −2.43 to 1.41; *p* = 0.60, Figure 7), indicating that acupuncture treatment could improve the Bristol Stool Scale score as compared to control samples.

### 3.7 Defecation Difficulty Score

**Figure 8:**
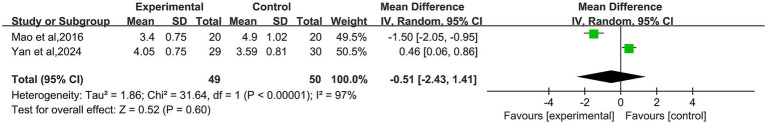
Forest plot of meta-analysis on Defecation Difficulty Score for functional constipation in elderly people treated by acupuncture.

Two articles reported acupuncture treatment for Defecation Difficulty Score (18, 22). A total of 99 patients were treated with heterogeneity (*p* = 0.17, *I*^2^ = 46%) and the outcome assessment indicator selected the fixed-effect model (MD: −0.19; 95% CI: −0.45–0.08; *p* = 0.16; Figure 8). The overall result indicated that the two groups showed no significant difference in alleviating the Defecation Difficulty Score.

### 3.8 Patient Assessment of Constipation Quality of Life

**Figure 9:**
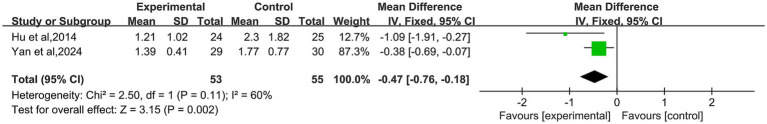
Analysis of Patient Assessment of Constipation Quality of Life through fixed-effects analysis model.

Two studies reported PAC-QOL (21, 22), having a total of 108 cases. Heterogeneity analysis suggested a small homogeneity in the research (*p* = 0.11, *I*^2^ = 60%). A fixed-effects model was selected to analyze the study data. Compared with the control group, the experimental group had no obvious advantage in changing the index (MD: −0.47; 95% CI: −0.76 to −0.18; *p* = 0.002, Figure 9).

### 3.9 Other secondary outcome assessment indicators

Only one article (20) reported the Clinic Constipation Score (CCS), plasma NOS, and plasma 5-HT. There was a significant difference in comparing the CCS between the two groups (*p* < 0.05). The NOS and 5-HT contents changed significantly after intervention in both groups (*p* < 0.05). In conclusion, acupuncture can improve NOS and 5-HT contents in the gastrointestinal tract. One study documented that acupuncture increased changes in bowel movements (23). An article (24) reported the improvement of TCM symptom scores was better in the treatment group than in the control group (*p* < 0.01).

### 3.10 Security analysis

In this study, only one study (23) reported the adverse effects of acupuncture in the treatment of functional constipation. Only one case was evaluated and the main symptoms were acupuncture dizziness, nausea, dizziness, diarrhea, and fatigue. None of the adverse reactions affected the treatment regimen in subsequent studies.

### 3.11 Publication bias

Because of the small number of articles included in our study, although no funnel plot was drawn to appraise publication bias, the results of the meta-analysis have no significant difference through the combined calculation of conversion effect models, showing that the research results were reliable.

## 4 Discussion

### 4.1 Summary of main results

This is the first systematic review and meta-analysis conducted based on RCTs to understand if acupuncture is beneficial for treating functional constipation in the elderly. This study assessed the treatment effectiveness of acupuncture as the primary treatment on the effective rate, complete spontaneous bowel movements, spontaneous bowel movements, the Bristol Stool Scale score, Defecation Difficulty Score, patient-assessment of constipation quality of life, the Clinic Constipation Score, plasma NOS, plasma 5-HT, changes in bowel movements, and traditional Chinese Medicine symptom scores for old people with functional constipation. Eight articles were adopted in the study, including 469 elderly with functional constipation. Through this study, we found that acupuncture can prominently improve the clinical treatment effect. Although each subgroup of the meta-analysis includes only two to four articles, this breakdown helps better illustrate the potential of acupuncture for treating functional constipation. Tianshu (ST25) is the abdominal point of the stomach channel and the fundraising point of the large intestine channel of the foot of the Yangming dynasty, and it is the most frequently selected point for the treatment of functional constipation. All the articles included in this study took Tianshu point (ST25) as one of the acupuncture points, and the results showed that Tianshu point (ST25) had a definite effect on functional constipation.

Meanwhile, compared with the control group, the treatment group had a statistically significant difference in complete spontaneous bowel movements after treatment. Two groups showed no significant difference in spontaneous bowel movements. Regarding the Bristol Stool Scale score, there is a significant difference. The Defecation Difficulty Scores and PAC-QOL had *p*-values that indicated no significant effect. However, acupuncture increased changes in bowel movements; there was a significant difference in comparing the CCS between the two groups. The NOS and 5-HT contents were changed significantly after intervention in both groups. An article reported that the improvement of TCM symptom scores was better in the treatment group than in the control group. The results suggested that acupuncture can achieve a good effect in the treatment of functional constipation; of course, in a few indicators, acupuncture also cannot significantly improve the corresponding symptoms.

So far, functional constipation has become a major public health problem all over the world (26). Modern medical research shows that the cause of functional constipation is related to pelvic floor muscle dysfunction, anal sphincter dysfunction, intestinal flora disorder, living habits, psychological factors, social environment, drug abuse, and other factors (27). Its pathogenesis is thought to be associated with intestinal nervous system disease, gastrointestinal motility disorder, Cajal mesenchymal cells, hormone neurotransmitter abnormalities, and gut-brain interaction abnormalities (28). Gastrointestinal dysfunction or decline and intestinal fluid secretion insufficiency are the main causes of functional constipation, most common in the elderly. However, with Western medical treatment, clinical symptoms can be temporarily controlled. But until now, there has been no particularly effective medication to cure functional constipation. In addition, Western medicine treatment of functional constipation has side effects and obvious limitations, which seriously affect the quality of life of the elderly. Based on this, acupuncture has a broader development space and prospects (29, 30). In recent years, the clinical research and pathological mechanism of acupuncture treatment of functional constipation have gradually increased, and increasing attention has been paid to acupuncture by the majority of medical personnel (30–32). A large number of clinical studies and animal experiments have found that both conventional manual acupuncture and electroacupuncture can enhance body immunity, improve constipation symptoms, and regulate gastrointestinal motility (33). Animal experimental research indicates that acupuncture stimulation of relevant acupoints in a slow transit constipation model in rats can increase the expression levels of glial cell-derived neurotrophic factor (GDNF) in colonic tissues, repair the ultramicrostructure of the colonic tissues, thereby improving the intestinal transit ability of the model rats, and effectively alleviating constipation symptoms (34).

Ning’s (35) research involves stimulating a specific acupoint in the constipation model mice through acupuncture, and it has been found that acupuncture can regulate the slow wave frequency and amplitude of the mouse colon, increase the number of interstitial cells of Cajal (ICC) in the colon, promote the upregulation of c-kit protein expression, and alleviate constipation symptoms. This suggests that acupuncture may have a therapeutic effect on constipation in elderly patients by modulating gastrointestinal neurotransmitters and enhancing intestinal motility. Zhang’s et al. (36) study suggests that acupuncture treatment for elderly functional constipation may improve intestinal motility by inhibiting certain neurotransmitters.

A substantial amount of research on the mechanisms of functional constipation indicates that acupuncture has a distinct advantage over the current mainstream Western medicine treatments, with significant effects (37).

Recent studies have indicated that in recent years, acupuncture has been proposed for managing bowel disorders in both infectious and oncological contexts, as well as for functional causes. Whenever possible, conservative strategies such as rehabilitation (38) or acupuncture (39) should be preferred over pharmacological treatments in these conditions to minimize side effects and enhance patient outcomes.

### 4.2 Strengths and limitations

Our study boasts several methodological strengths, including its rigorous blinding procedures, multi-center design, and the robustness conferred by a large sample size, all underpinned by a meticulously executed concealed randomization process. This robust framework ensures the reliability and generalizability of our findings. Despite the methodological rigor of our study, it is not without its limitations. Firstly, the scope of our review was constrained by the inclusion of only eight literature sources, which were predominantly limited to domestic studies and did not encompass international research. This resulted in a modest sample size and a narrow demographic focus, as all participants were elderly individuals. Secondly, the overall quality of the literature assessed was found to be suboptimal. The outcome indicators were not exhaustive, omitting critical parameters such as medical costs, long-term efficacy, and follow-up duration. These omissions limit the comprehensiveness of our findings and the depth of insight into the broader implications of the treatment. Thirdly, we identified inconsistencies in the Western medical treatment protocols reported in one of the studies, which could introduce operational bias and potentially skew the effectiveness of the results. Additionally, the selection of acupuncture points and the execution of techniques are inherently subjective, which may engender variability in treatment efficacy across different practitioners.

## 5 Conclusion

Acupuncture has demonstrated efficacy in alleviating functional constipation among the elderly, notably enhancing the treatment’s effectiveness. For practitioners of acupuncture in clinical settings, the available evidence substantiates the inclusion of acupuncture as a viable therapeutic option for managing functional constipation. While acupuncture shows promise, the current evidence is of low to moderate quality, and more rigorous studies are needed. Looking ahead, it is imperative that the caliber of research pertaining to acupuncture’s role in treating functional constipation be continually refined. Additionally, in the future, research should focus on the therapeutic effects of specific meridians and different acupoints. Greater emphasis should be placed on investigating the underlying mechanisms involving gastrointestinal hormones, neurotransmitter levels, and intestinal motility functions, as these areas are pivotal to understanding the therapeutic impact of acupuncture. Furthermore, there is a need for future studies to employ larger sample sizes and to adhere to rigorous, placebo-controlled methodologies. Incorporating a diverse array of outcome indicators will not only bolster the robustness of the findings but also augment the applicability and practicality of the conclusions drawn from such research endeavors. This comprehensive approach will pave the way for more informed clinical decisions and enhanced patient care in the realm of functional constipation treatment.

## Data Availability

The original contributions presented in the study are included in the article/Supplementary material, further inquiries can be directed to the corresponding author.
