# Supplementary material for: Efficacy of acupuncture for functional constipation in elderly: a systematic review and meta-analysis
Source: Front Med (Lausanne). 2024 Dec 4;11:1473847. doi: 10.3389/fmed.2024.1473847 (PMC11652177; doi:10.3389/fmed.2024.1473847)
Supplement: Supplementary file 1 [file Data_Sheet_1.docx]

Supplementary Material

## TABLES

TABLE 1: PubMed: session results.

| **Number** | **Query** | **Search Details** | **Results** |
| --- | --- | --- | --- |
| #8 | #3 AND #6 | (("Constipation"[MeSH Terms] OR "Constipation"[Title/Abstract]) AND ("Acupuncture"[MeSH Terms] OR ("Pharmacopuncture"[Title/Abstract] OR "acupuncture treatment"[Title/Abstract] OR "needling"[Title/Abstract] OR ("acupunture"[All Fields] AND "therapy"[Title/Abstract]) OR "needling therapy"[Title/Abstract]))) AND (clinicaltrial[Filter] OR randomizedcontrolledtrial[Filter]) | 17 |
| #7 | #3 AND #6 | ("Constipation"[MeSH Terms] OR "Constipation"[Title/Abstract]) AND ("Acupuncture"[MeSH Terms] OR ("Pharmacopuncture"[Title/Abstract] OR "acupuncture treatment"[Title/Abstract] OR "needling"[Title/Abstract] OR ("acupunture"[All Fields] AND "therapy"[Title/Abstract]) OR "needling therapy"[Title/Abstract])) | 67 |
| #6 | #4 OR #5 | "Acupuncture"[MeSH Terms] OR ("Pharmacopuncture"[Title/Abstract] OR "acupuncture treatment"[Title/Abstract] OR "needling"[Title/Abstract] OR ("acupunture"[All Fields] AND "therapy"[Title/Abstract]) OR "needling therapy"[Title/Abstract]) | 9594 |
| #5 | ((((Pharmacopuncture[Title/Abstract]) OR (Acupuncture treatment[Title/Abstract])) OR (needling[Title/Abstract])) OR (acupunture therapy[Title/Abstract])) OR (needling therapy[Title/Abstract]) | "Pharmacopuncture"[Title/Abstract] OR "acupuncture treatment"[Title/Abstract] OR "needling"[Title/Abstract] OR ("acupunture"[All Fields] AND "therapy"[Title/Abstract]) OR "needling therapy"[Title/Abstract] | 7886 |
| #4 | "Acupuncture"[Mesh] | "Acupuncture"[MeSH Terms] | 2094 |
| #3 | #1 OR #2 | "Constipation"[MeSH Terms] OR "Constipation"[Title/Abstract] | 36058 |
| #2 | constipation[Title/Abstract] | "constipation"[Title/Abstract] | 31698 |
| #1 | "Constipation"[Mesh] | "Constipation"[MeSH Terms] | 16709 |

TABLE 2: characteristics of 8 studies.

| **Study** | **Study**  **design** | **Sample**  **size**  **(T:C)** | **Age(year),**  **Mean±SD** | **Disease**  **duration(year),**  **Mean±SD** | **Sex**  **(M:F)** | **Intervention** | **Comparison** | **Treatment**  **duration**  **(days)** | **Follow-up**  **(months)** | **Outcomes** |
| --- | --- | --- | --- | --- | --- | --- | --- | --- | --- | --- |
| Mao et al,2016 | RCT | T:20  C:20 | T:62.85士2.71  C:56.95士9.83 | T:3.69士2.42  C:3.90士2.75 | (T)6:14  (C)8:12 | Electro-acupuncture | Prucalopride Succinate Tablets | 56 | NM | CSBMs,SBMs,Bristol Stool Scale score,defecation difficulty score |
| Cao et al,2012 | RCT | T:21  C:20 | T:51.2士7.23  C:52.4士7.65 | T:9.83士6.21  C:10.2士9.7 | (T)9:12  (C)8:12 | Electro-acupuncture | senna leaf | 17 | NM | the effective rate |
| Xu et al,2015 | RCT | T:30  C:30 | T:53士15  C:53士12 | T:3.79士3.64  C:4.03士3.28 | (T)12:18  (C)11:19 | Electro-acupuncture | Sham-electroacupuncture | 28 | NM | the effective rate,the Clinic Constipation Score,plasma NOS, plasma 5-HT |
| Hu et al,  2014 | RCT | T:24  C:25 | T:52.75±18.9  C:50.96±14.8 | T:12.23±11.98  C:13.61±14.07 | (T)9:15  (C)6:19 | Electro-acupuncture | Sham-electroacupuncture | 28 | NM  (Continued) | CSBMs,PAC-QOL |

| **Study** | **Study**  **design** | **Sample**  **size**  **(T:C)** | **Age(year),**  **Mean±SD** | **Disease**  **duration(year),**  **Mean±SD** | **Sex**  **(M:F)** | **Intervention** | **Comparison** | **Treatment**  **duration**  **(days)** | **Follow-up**  **(months)** | **Outcomes** |
| --- | --- | --- | --- | --- | --- | --- | --- | --- | --- | --- |
| Yan et al,2024 | RCT | T:29  C:30 | T:62.79士9.77  C:59.73士8.49 | T:149.59士109.89  C:132.80士96.05 | (T)0:29  (C)0:30 | acupuncture | Sham-acupuncture | 16 | NM | SBMs,Bristol Stool Scale score,defecation difficulty score,PAC-QOL |
| Li et al,2021 | RCT | T:40  C:40 | T:58.06士12.12  C:57.78士11.54 | T:7.19士5.96  C:47.7士11.54 | (T)18:22  (C)19:21 | Electro-acupuncture | senna leaf | 40 | NM | changes in bowel movements |
| Sun et al,2016 | RCT | T:40  C:40 | T:60.1士12.4  C:61.1士13.6 | T:1.8士0.6  C:1.7士0.8 | (T)15:25  (C)12:28 | acupuncture | Mosapride citrate dispersible tablets | 8 | NM | the effective rate,Traditional Chinese Medicine (TCM) symptom scores |
| Fu et al,2012 | RCT | T:30  C:30 | T:74.3士2.71  C: 73.5士7.1 | T:6.8士4.2  C:6.5士0.7 | (T)12:18  (C)13:17 | acupuncture | Maren Runchang Wan | 20 | NM | the effective rate |

TABLE 2 (Continued)

Note: T, intervention group; C, comparison group; M, male; F, female; NM, not mention,; RCT, randomized controlled trial; CSBMs, complete spontaneous bowel movements; SBMs, spontaneous bowel movements; PAC-QOL, Patient-assessment of constipation quality of life.
